# Analytical Biases Associated with GC-Content in Molecular Evolution

**DOI:** 10.3389/fgene.2017.00016

**Published:** 2017-02-15

**Authors:** Jonathan Romiguier, Camille Roux

**Affiliations:** Department of Ecology and Evolution, University of LausanneLausanne, Switzerland

**Keywords:** GC-content, positive selection, biased gene conversion, codon usage bias, phylogeny, methodological biases

## Abstract

Molecular evolution is being revolutionized by high-throughput sequencing allowing an increased amount of genome-wide data available for multiple species. While base composition summarized by GC-content is one of the first metrics measured in genomes, its genomic distribution is a frequently neglected feature in downstream analyses based on DNA sequence comparisons. Here, we show how base composition heterogeneity among loci and taxa can bias common molecular evolution analyses such as phylogenetic tree reconstruction, detection of natural selection and estimation of codon usage. We then discuss the biological, technical and methodological causes of these GC-associated biases and suggest approaches to overcome them.

## Introduction

GC-content is shaped by a complex balance among mutation, selection, recombination, and genetic drift (Bulmer, 1991; Eyre-Walker and Hurst, 2001; Duret et al., 2002). As a consequence of variation in this subtle balance, it has been observed that GC-content varies considerably at two levels: (i) among genomes from different species and (ii) along chromosomes of a single species (Bernardi et al., 1985). Among species, the average genomic GC-content ranges from 13 to 75% (Pagani et al., 2011). Within the same genome, large chromosomal regions can also greatly differ in their nucleotide composition as first described in humans (Bernardi et al., 1985). For instance, GC-content is distributed across the human genome over successive long stretches of >100 kb that can be either GC-rich (with a GC-content ∼60%) or GC-poor (with a GC-content ∼35%; International Human Genome Sequencing Consortium, 2001).

**FIGURE 1:**
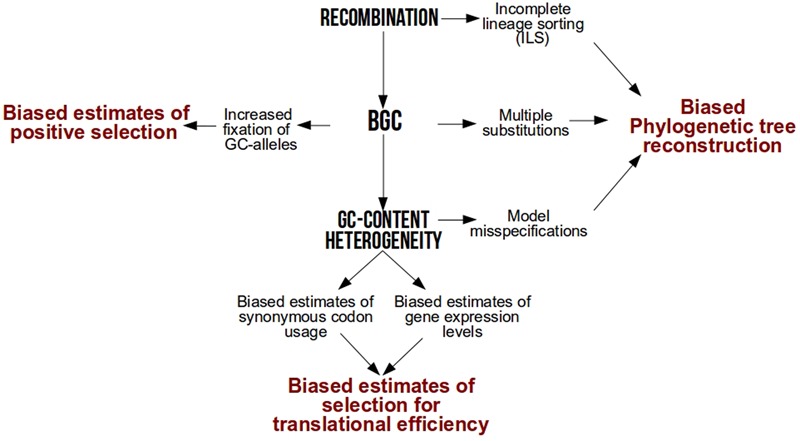
**Methodological biases associated to recombination, GC-biased gene conversion (BGC) and GC-content heterogeneity**.

After several years of debate among neutral or selective hypotheses [reviewed in Duret and Galtier (2009)], it is now widely accepted that one of the major drivers of base composition heterogeneity is GC-biased gene conversion (gBGC), a repair bias that favors GC over AT alleles during meiotic recombination (Eyre-Walker, 1993; Galtier et al., 2001; Montoya-Burgos et al., 2003; Duret and Arndt, 2008; Kent et al., 2012; Arbeithuber et al., 2015; Mugal et al., 2015). As a result of this link between GC and recombination, local GC-content increases faster in genomic hotspots of recombination (Spencer, 2006) while genome-wide GC-content increases faster in species with higher recombination rates per time unit (Romiguier et al., 2010, 2013b; Figuet et al., 2014; Weber et al., 2014). By conferring a higher transmission probability of GC alleles over AT in heterozygotes, gBGC mimics natural selection but is frequently overlooked in molecular evolution studies. Here, we revisit how much intra-genomic and inter-specific variations in base composition have a strong power to bias popular analyses in molecular evolution such as phylogenetic tree reconstruction, detection of natural selection and estimation of codon usage bias (**Figure 1**).

## Phylogenetic Tree Reconstruction

Reconstructions of phylogenetic trees from molecular datasets are central in evolutionary biology. While initially limited to a handful of loci with limited power to resolve difficult phylogenetic relationships, phylogenetic tree reconstruction is no longer restricted by the number of genetic markers. However, some phylogenetic relationships of the Tree of Life remain unresolved (Philippe et al., 2011). This difficulty stems from the mosaic nature of genomes gathering alternative and conflicting gene trees (Degnan and Rosenberg, 2009), where some but not all loci support the true species genealogy. Determining which loci are reliable phylogenetic markers is thus one of the biggest challenges in phylogenomics. While mixed historical signals along genomes are likely to have different natures, we will here focus to issues related to base composition.

A recent phylogenomic study reported that base composition is a relevant criterion to select markers carrying unambiguous phylogenetic signal: gene GC-content (average GC% of the sequences of an alignment) and GC-heterogeneity (variance of GC% among sequences of an alignment) were proved to bias species tree reconstructions (Romiguier et al., 2013a). As illustrated with mammalian genomes, phylogenetic trees of genes located in GC-rich regions produce five times more contradicting topologies than GC-poor genes, leading to important reconstruction biases and a poor resolution for both accepted and controversial nodes. This negative analytical effects of GC-content on tree reconstructions is widespread across the tree of life, as reported in basal eukaryote lineages (Rodríguez-Ezpeleta et al., 2007), yeasts (Collins et al., 2005), beetles (Sheffield et al., 2009), bees (Romiguier et al., 2016), hexapods (Delsuc, 2003), fishes (Li and Ortí, 2007; Betancur-R et al., 2013), birds (Nabholz et al., 2011), and bats (Teeling et al., 2000). Despite the accumulating empirical evidence demonstrating the pervasiveness of base composition issues in phylogeny, the reasons underlying such strong biases are unexplored. Here, we suggest three non-mutually exclusive hypotheses to explain this negative GC-effect in phylogenomics studies.

First, some aspects of the GC-bias are likely to be due to model misspecifications. Probabilistic methods for phylogenetic reconstruction (maximum likelihood or Bayesian inference) are indeed generally based on models of sequence evolution that assume a homogeneous base composition along the tree. However, this assumption is often violated (Phillips et al., 2004). Indeed, average GC-content of an alignment correlates strongly with GC-heterogeneity among sequences as a result of variation in the dynamic of gBGC among sampled species (Romiguier et al., 2013a). Such departures from the assumption of base composition homogeneity can lead to severe biases by incorrectly grouping distantly related taxa that converge in extreme nucleotide composition on a given locus (Phillips et al., 2004). This type of issues can be, however, easily solved by model-based solutions (see last paragraph of this section for more details).

The second hypothesis proposes that incomplete lineage sorting (ILS) is more important in GC-rich than GC-poor regions. ILS is known to produce conflicts among gene trees and the species tree because of the retention of ancestral polymorphisms (Degnan and Rosenberg, 2009). At the scale of the whole genome, the amount of incompletely sorted genes increases when the time of divergence is small relative to the average effective population sizes (Clark, 1997). Genomic variation in ILS was also empirically reported to be associated to GC-content in hominid genomes (Hobolth et al., 2011). This indirect (Charlesworth et al., 1993) relationship between GC-content and ILS can be explained by the dual effects of local recombination rates on base composition and linkage disequilibrium. High local recombination rates increase GC-content through gBGC, but also decrease the effect of genetic interferences, i.e., background selection (Charlesworth et al., 1993) and hitchhiking (Smith and Haigh, 1974). By being less affected by linked selective processes, GC-rich regions are thus expected to have relatively higher effective population sizes than GC-poor regions, leading to an extended retention time of ancestral polymorphism.

The third hypothesis is that gBGC is associated to saturation in multiple substitutions. Following the rapid birth and death of local recombination hotspots, gBGC is expected to occur in short, intense episodes (Duret and Galtier, 2009) where deleterious GC substitutions are likely to occur (Necşulea et al., 2011). Following a gBGC episode, natural selection is likely to revert such deleterious substitutions through AT replacement (Galtier et al., 2009). This toggling between GC deleterious and AT compensatory substitutions at the same nucleotide site is expected to lead to homoplasy, a direct consequence of multiple substitutions causing spurious similarity not due to common ancestry (Philippe et al., 2011). This type of AT/GC toggling is expected to be particularly fast and difficult to track because of the short-life of gBGC episodes that depends on the self-destructive nature of recombination hotspots (Coop and Myers, 2007). Even at very short evolutionary scales such as the Denisovan/Modern human divergence (0.4–0.8 Myrs), local recombination hotspots are not conserved (Lesecque et al., 2014), which could imply a complete loss of phylogenetic signals due to multiple turnovers between gBGC and natural selection at larger evolutionary scales. Although genomically small (1–2 kb), these short-lived recombination hotspots tend to arise and disappear in the same genomic regions of 1–2 Mb (Duret and Galtier, 2009) exhibit homoplasy issues. Common in fast-evolving sequences, homoplasy is also at the origin of the so-called and undesired “long branch attraction artifact” (Felsenstein, 1978). Reinforcing the idea that GC-rich genes might be affected by such biases, GC-rich and GC-heterogeneous genes have fast rates of evolution (Romiguier et al., 2013a, 2016). These abnormally fast-evolving genes are then likely to cause long-branch attraction artifacts, but also more general issues related to heterotachy-driven biases (Philippe et al., 2005). Even if long-branch attraction is generally considered as a minor problem in likelihood-based phylogenetics compared to parsimony, maximum likelihood methods using GC-rich genes can have a biased support toward topologies grouping long branches together (Romiguier et al., 2013a, 2016).

One solution to cope with base composition issues is the use of models of sequence evolution that takes into account heterogeneity in GC-content (Galtier and Gouy, 1998; Foster, 2004; Blanquart and Lartillot, 2006; Boussau and Gouy, 2006; Gowri-Shankar and Rattray, 2007; Dutheil and Boussau, 2008). However, these so-called non-homogeneous models are computationally costly. Albeit useful to alleviate GC-heterogeneity issues in phylogeny, empirical studies illustrate their limits to retrieve high bootstrap supports in the most GC-heterogeneous sequences (Betancur-R et al., 2013; Romiguier et al., 2016), shedding light on other GC-dependent biases in phylogeny such as ILS and gBGC-driven homoplasy. To date, the best practice recommended to discard noisy signals in sequences is the use of non-homogeneous models and/or the use of GC-poor phylogenetic markers. In this regard, it is noteworthy that coding sequences tend to be clustered in recombination hotspots and GC-rich regions (Duret and Galtier, 2009). Consequently, the use of the rare phylogenetic markers located in AT-rich regions is recommended. This is the case of ultra-conserved non-coding elements (UCE) that have the advantage to be AT-rich and evolve particularly slowly (McCormack et al., 2012). Compared to these non-coding AT-rich markers, clusters of AT-rich coding genes in low-recombining regions could undergo a higher rate of background selection, decreasing the effective population size and then, the amount of ILS. It is noteworthy that UCE and AT-rich genes both support the same topology for the controversial rooting of placental mammals (McCormack et al., 2012; Romiguier et al., 2013a), highlighting relevance of these markers to overcome GC-biases. Other strategies might involve to compare these markers with markers that cannot be affected by recombination and gBGC, such as mitochondrial genes. Further methodological improvements could come from coalescent-based supertree methods (Liu et al., 2009) that account for ILS. By weighting the confidence in each gene tree according to the GC-content of an alignment, they may allow the integration of most of the available information and alleviate the spurious signal inherent to GC-rich markers. To date, methods computing the exact likelihood of alternative topologies are restricted to relatively simple models neglecting direct and indirect effects of background selection, selective sweep, gBGC and ILS on phylogenetic reconstruction. But these processes are now implemented in recent simulators (Haller and Messer, 2017), allowing them to be treated as nuisance parameters during computational evolution of sequences. Although such highly complex models are currently intractable by maximum likelihood approaches, the possibility to simulate them within an approximate Bayesian computation (ABC) framework (Beaumont et al., 2002; Csilléry et al., 2010a,b; Pudlo et al., 2016) could bring new methodological perspectives in phylogenetic reconstruction. ABC has been proved to be a powerful framework to compare complex evolutionary scenarios for large datasets (Roux et al., 2016), illustrating the recent improvements made in flexible machine learning algorithms. Applied to phylogenetic reconstructions, efficient computational tools like SLiM 2 are already available to simulate models with gBGC episodes and multiple substitutions along a branch as well as statistical packages to compute the probabilities of alternative scenarios (Csilléry et al., 2012; Pudlo et al., 2016). Altogether, current available softwares already provide stimulating leads for future developments in phylogeny.

## Detection of Positive Selection

Identifying candidate loci for natural selection is a central goal explored by two traditional approaches in adaptation-genomics: top-down (GWA and QTL) and bottom-up (genomic scan) approaches. With the advent of high-throughput sequencing, genomic scans became a popular approach to detect candidate target of selection. Such scans have the merit to identify candidates without the *a priori* expectation of a candidate gene approach (Ellegren, 2014). However, they have various limitations with false-positive issues (Mallick et al., 2009; Bierne et al., 2011), narrow signatures of balancing selection (Roux et al., 2012), and over-interpretation of outlier loci (Pavlidis et al., 2012). Here, we detail how GC-content can lead to important additional bias during genome scans for detecting natural selection.

Genome scans of positive selection often rely on methods that look for lineage-specific accelerations in the protein rate of evolution. Such accelerations are classically measured through *dN/dS*, which calculates the excess of amino-acid substitutions (*dN*: non-synonymous mutation rate per site) relative to *dS*, the substitution rate per site used as a proxy of the neutral clock. This *dN/dS* ratio is generally smaller than 1, reflecting the pervasiveness of purifying selection that eliminates non-synonymous mutations to preserve the protein structure. Conversely, a *dN/dS* ratio greater than 1 is considered as a signature of positive selection that favors the fixation of beneficial non-synonymous mutations. From a population genetics point of view, gBGC mimics positive selection by favoring the fixation of AT- > GC mutations, regardless of their beneficial or deleterious status (Nagylaki, 1983). Because GC alleles are actively selected by the repair systems of meiotic recombination, they are over-represented in the gamete pool and benefit of increased transmission to the next generation in a similar way than beneficial mutations subject to positive selection. Consequently, many accelerations of the substitution rate attributed to positive selection during genome scans are actually due to gBGC episodes (Galtier and Duret, 2007; Berglund et al., 2009; Galtier et al., 2009; Ratnakumar et al., 2010; Kostka et al., 2012). When a mutation toward GC is deleterious, gBGC can counteract positive selection and maintain or fix deleterious alleles. High fixation rates of non-synonymous mutations at a locus should thus not be systematically interpreted as being beneficial for the fitness of the individual, particularly when considering that gBGC has been proved to be able to maintain deleterious mutations associated to human diseases (Necsulea et al., 2011; Capra et al., 2013; Lachance and Tishkoff, 2014).

Confusion between positive selection and gBGC could be avoided through two different ways. The first is by filtering the results of classical tests of positive selection and consider with caution positive selection signatures in GC-rich regions. This is particularly true for selection tests that rely more on overall evolutionary rate rather than *dN/dS* (Pollard et al., 2006; Kostka et al., 2012). Even if gBGC can increase *dN/dS* in some conditions (Galtier et al., 2009; Bolívar et al., 2015), AT- > GC mutations are more likely to happen in synonymous sites, which limits the effect of gBGC on *dN/dS* compared to the evolutionary rate. Several criterions can be used in both cases to differentiate gBGC from positive selection, such as the number of mutations toward GC in the surrounding non-coding regions (Galtier and Duret, 2007). The second would be to develop methods that restrict *dN/dS* estimations to GC-conservative substitutions in the context of codon-models aimed to detect positive selection events (Yang and Nielsen, 2002; Lartillot, 2013).

## Codon Usage Bias

Popular analytical methods in molecular evolution rely on a strong assumption: synonymous mutations are neutral. GC-content at synonymous positions is frequently claimed to be exposed only to the mutation/drift equilibrium. However, natural selection was proposed to be superimposed to these two evolutionary forces at synonymous codons (Urrutia, 2003; Comeron, 2004; Plotkin et al., 2004). Although initially challenged (Williamson et al., 2005), natural selection acting on standing synonymous variation was found to be associated to gene expression level, the most expressed genes using a set of preferred codons (Comeron, 2004). This association is explained by selection for increased translational efficiency. The analysis of >1,000 genes in *Drosophila* demonstrated that the most used synonymous codons corresponded to the most available tRNAs in the genome (Moriyama and Powell, 1997). Translational efficiency would then be optimized by increasing the usage of the preferred synonymous codons. Such a process can be tested in coding sequences by measuring the effective number of codons (ENc) in a given gene. ENc takes a value of 61 when all codons of the genetic code (minus the three stop codons) are used without bias, and decreases to 20 (the number of amino-acids) for the most biased genes. In agreement with the hypothesis of selection for translational efficiency, population genetics analyses in *Drosophila* described signatures of selection on synonymous mutations (Akashi, 1995; Akashi and Schaeffer, 1997).

A study of codon usage bias in *Caenorhabditis elegans, Drosophila melanogaster*, and *Arabidopsis thaliana* has shed light on the over-expression of genes featuring codon preference, with a large predominance of preferred codons ending with G or C (Duret and Mouchiroud, 1999). However, the GC-content at third coding positions (GC3) is also correlated to the GC-content of the surrounding non-coding regions (Kliman and Hey, 1994; Akashi et al., 1998), which suggests the action of gBGC shaping local base compositions. By locally increasing GC-content, gBGC mechanically restricts the number of used codons and reduces the measured ENc independently of selection for translational efficiency. The measured ENc is thus biased by gBGC and must be corrected with local background nucleotide compositions. In addition, variation in GC-content also impacts measures of gene expression. With the advent of high-throughput sequencing technologies, it is now a standard practice to approximate gene expression levels by counting the number of reads mapping a target in ChIP-seq or RNA-seq analysis. However, sequencing biases artificially over-represent genomic regions with intermediate levels of GC-content (50%), which in turn bias the estimates of gene expression levels (Chouvarine et al., 2016). Testing selection for translational efficiency by measuring the correlation between ENc and gene expression levels therefore requires the use of both GC-corrected ENc and GC-corrected expression levels. ENc estimates can be corrected by GC-content of neighborhood regions (Novembre, 2002), while GC-corrected expression levels can be obtained by applying local LOESS regression (Miller et al., 2011; Benjamini and Speed, 2012; Chandrananda et al., 2014) or quantile normalization-methods (Risso et al., 2011), i.e., by normalizing the raw number of mapped reads by the local GC-content.

The ongoing surge of transcriptomic data will permit measurement of GC-content heterogeneity, preferred codons usage and expression levels across a large number of loci and species. This type of large-scale analysis could open the door to a better understanding of the relationship linking effective population sizes (*Ne*) and codon usage. As theoretically predicted (Bulmer, 1991), selection on synonymous codons might be stronger in species with large *Ne*. While the *Ne*-hypothesis to explain variation in selection on codon usage remains untested by empirical studies, a descriptive study of the *Ne*-effect on variation in gBGC will be necessary to avoid entangling the two effects. Future projects aiming to test these hypotheses are expected to be strongly biased if GC-content biases are naively neglected regarding estimates of gene expression levels or codon usage.

## Conclusion

GC-content is associated to multiple biases of different nature (**Figure 1**). Whether through technological reasons (sequencing technologies biases), biological reasons (GC-biased gene conversion) or methodological reasons (models of sequence evolution limitations), all these biases affect the results of downstream analyses. With the surge of genomic data from various non-model species, comparative genomics have the opportunity to solve many unresolved questions in evolution. However, one should be aware of the methodological challenges associated to the GC-content heterogeneity inherent to large scale studies, whether it be for a large number of species or loci.

## Author Contributions

JR had the idea of the project. JR and CR wrote the article.

## Conflict of Interest Statement

The authors declare that the research was conducted in the absence of any commercial or financial relationships that could be construed as a potential conflict of interest.

## References

[B1] Akashi H. (1995). Inferring weak selection from patterns of polymorphism and divergence at “silent” sites in *Drosophila* DNA. *Genetics* 139 1067–1076. 7713409 10.1093/genetics/139.2.1067 PMC1206357

[B2] Akashi H. Kliman R. M. Eyre-Walker A. (1998). Mutation pressure, natural selection, and the evolution of base composition in *Drosophila*. *Genetica* 10 49–60. 10.1023/A:1017078607465 9720271

[B3] Akashi H. Schaeffer S. W. (1997). Natural selection and the frequency distributions of “silent” DNA polymorphism in *Drosophila*. *Genetics* 146 295–307. 9136019 10.1093/genetics/146.1.295 PMC1207945

[B4] Arbeithuber B. Betancourt A. J. Ebner T. Tiemann-Boege I. (2015). Crossovers are associated with mutation and biased gene conversion at recombination hotspots. *Proc. Natl. Acad. Sci. U.S.A.* 112 2109–2114. 10.1073/pnas.1416622112 25646453 PMC4343121

[B5] Beaumont M. A. Zhang W. Balding D. J. (2002). Approximate Bayesian computation in population genetics. *Genetics* 162 2025–2035. 12524368 10.1093/genetics/162.4.2025 PMC1462356

[B6] Benjamini Y. Speed T. P. (2012). Summarizing and correcting the GC content bias in high-throughput sequencing. *Nucleic Acids Res.* 40:e72 10.1093/nar/gks001 PMC3378858 22323520

[B7] Berglund J. Pollard K. S. Webster M. T. (2009). Hotspots of biased nucleotide substitutions in human genes. *PLoS Biol.* 7:e26 10.1371/journal.pbio.1000026 PMC2631073 19175294

[B8] Bernardi G. Olofsson B. Filipski J. Zerial M. Salinas J. Cuny G. (1985). The mosaic genome of warm-blooded vertebrates. *Science* 228 953–958. 10.1126/science.4001930 4001930

[B9] Betancur-R R. Li C. Munroe T. A. Ballesteros J. A. Ortí G. (2013). Addressing gene tree discordance and non-stationarity to resolve a multi-locus phylogeny of the flatfishes (Teleostei: Pleuronectiformes). *Syst. Biol.* 62 763–785. 10.1093/sysbio/syt039 23749787

[B10] Bierne N. Welch J. Loire E. Bonhomme F. David P. (2011). The coupling hypothesis: why genome scans may fail to map local adaptation genes. *Mol. Ecol.* 20 2044–2072. 10.1111/j.1365-294X.2011.05080.x 21476991

[B11] Blanquart S. Lartillot N. (2006). A Bayesian compound stochastic process for modeling nonstationary and nonhomogeneous sequence evolution. *Mol. Biol. Evol.* 23 2058–2071. 10.1093/molbev/msl091 16931538

[B12] Bolívar P. Mugal C. F. Nater A. Ellegren H. (2015). Recombination rate variation modulates gene sequence evolution mainly via GC-biased gene conversion, not Hill–Robertson interference, in an avian system. *Mol. Biol. Evol.* 33 216–227. 10.1093/molbev/msv214 26446902 PMC4693978

[B13] Boussau B. Gouy M. (2006). Efficient likelihood computations with nonreversible models of evolution. *Syst. Biol.* 55 756–768. 10.1080/10635150600975218 17060197

[B14] Bulmer M. (1991). The selection-mutation-drift theory of synonymous codon usage. *Genetics* 129 897–907. 1752426 10.1093/genetics/129.3.897 PMC1204756

[B15] Capra J. A. Hubisz M. J. Kostka D. Pollard K. S. Siepel A. (2013). A model-based analysis of GC-biased gene conversion in the human and chimpanzee genomes. *PLoS Genet.* 9:e1003684 10.1371/journal.pgen.1003684 PMC3744432 23966869

[B16] Chandrananda D. Thorne N. P. Ganesamoorthy D. Bruno D. L. Benjamini Y. Speed T. P. (2014). Investigating and correcting plasma DNA sequencing coverage bias to enhance aneuploidy discovery. *PLoS ONE* 9:e86993 10.1371/journal.pone.0086993 PMC3906086 24489824

[B17] Charlesworth B. Morgan M. T. Charlesworth D. (1993). The effect of deleterious mutations on neutral molecular variation. *Genetics* 134 1289–1303. 8375663 10.1093/genetics/134.4.1289 PMC1205596

[B18] Chouvarine P. Wiehlmann L. Losada P. M. DeLuca D. S. Tümmler B. (2016). Filtration and normalization of sequencing read data in whole-metagenome shotgun samples. *PLoS ONE* 11:e0165015 10.1371/journal.pone.0165015 PMC5070866 27760173

[B19] Clark A. G. (1997). Neutral behavior of shared polymorphism. *Proc. Natl. Acad. Sci. U.S.A.* 94 7730–7734. 10.1073/pnas.94.15.7730 9223256 PMC33687

[B20] Collins T. M. Fedrigo O. Naylor G. J. P. (2005). Choosing the best genes for the job: the case for stationary genes in genome-scale phylogenetics. *Syst. Biol.* 54 493–500. 10.1080/10635150590947339 16012114

[B21] Comeron J. M. (2004). Selective and mutational patterns associated with gene expression in humans: influences on synonymous composition and intron presence. *Genetics* 167 1293–1304. 10.1534/genetics.104.026351 15280243 PMC1470943

[B22] Coop G. Myers S. R. (2007). Live hot, die young: transmission distortion in recombination hotspots. *PLoS Genet.* 3:e35 10.1371/journal.pgen.0030035 PMC1817654 17352536

[B23] Csilléry K. Blum M. G. B. Gaggiotti O. E. François O. (2010a). Approximate Bayesian Computation (ABC) in practice. *Trends Ecol. Evol.* 25 410–418. 10.1016/j.tree.2010.04.001 20488578

[B24] Csilléry K. Blum M. G. B. Gaggiotti O. E. François O. (2010b). Invalid arguments against ABC: reply to A.R. Templeton. *Trends Ecol. Evol.* 25 490–491. 10.1016/j.tree.2010.06.011 20488578

[B25] Csilléry K. François O. Blum M. G. B. (2012). abc: an R package for approximate Bayesian computation (ABC). *Methods Ecol. Evol.* 3 475–479. 10.1111/j.2041-210X.2011.00179.x 20488578

[B26] Degnan J. H. Rosenberg N. A. (2009). Gene tree discordance, phylogenetic inference and the multispecies coalescent. *Trends Ecol. Evol.* 24 332–340. 10.1016/j.tree.2009.01.009 19307040

[B27] Delsuc F. (2003). Comment on “Hexapod origins: monophyletic or paraphyletic?” *Science* 301 1490–1491. 10.1126/science.1086558 12970547

[B28] Duret L. Arndt P. F. (2008). The impact of recombination on nucleotide substitutions in the human genome. *PLoS Genet.* 4:e1000071 10.1371/journal.pgen.1000071 PMC2346554 18464896

[B29] Duret L. Galtier N. (2009). Biased gene conversion and the evolution of mammalian genomic landscapes. *Annu. Rev. Genomics Hum. Genet.* 10 285–311. 10.1146/annurev-genom-082908-150001 19630562

[B30] Duret L. Mouchiroud D. (1999). Expression pattern and, surprisingly, gene length shape codon usage in *Caenorhabditis, Drosophila*, and *Arabidopsis*. *Proc. Natl. Acad. Sci. U.S.A.* 96 4482–4487. 10.1073/pnas.96.8.4482 10200288 PMC16358

[B31] Duret L. Semon M. Piganeau G. Mouchiroud D. Galtier N. (2002). Vanishing GC-rich isochores in mammalian genomes. *Genetics* 162 1837–1847. 12524353 10.1093/genetics/162.4.1837 PMC1462357

[B32] Dutheil J. Boussau B. (2008). Non-homogeneous models of sequence evolution in the Bio++ suite of libraries and programs. *BMC Evol. Biol.* 8:255 10.1186/1471-2148-8-255 PMC2559849 18808672

[B33] Ellegren H. (2014). Genome sequencing and population genomics in non-model organisms. *Trends Ecol. Evol.* 29 51–63. 10.1016/j.tree.2013.09.008 24139972

[B34] Eyre-Walker A. (1993). Recombination and mammalian genome evolution. *Proc. R. Soc. B Biol. Sci.* 252 237–243. 10.1098/rspb.1993.0071 8394585

[B35] Eyre-Walker A. Hurst L. D. (2001). The evolution of isochores. *Nat. Rev. Genet.* 2 549–555. 10.1038/35080577 11433361

[B36] Felsenstein J. (1978). Cases in which parsimony or compatibility methods will be positively misleading. *Syst. Zool.* 27 401–410. 10.2307/2412923

[B37] Figuet E. Ballenghien M. Romiguier J. Galtier N. (2014). Biased gene conversion and GC-content evolution in the coding sequences of reptiles and vertebrates. *Genome Biol. Evol.* 7 240–250. 10.1093/gbe/evu277 25527834 PMC4316630

[B38] Foster P. (2004). Modeling compositional heterogeneity. *Syst. Biol.* 53 485–495. 10.1080/10635150490445779 15503675

[B39] Galtier N. Duret L. (2007). Adaptation or biased gene conversion? Extending the null hypothesis of molecular evolution. *Trends Genet.* 23 273–277. 10.1016/j.tig.2007.03.011 17418442

[B40] Galtier N. Duret L. Glémin S. Ranwez V. (2009). GC-biased gene conversion promotes the fixation of deleterious amino acid changes in primates. *Trends Genet.* 25 1–5. 10.1016/j.tig.2008.10.011 19027980

[B41] Galtier N. Gouy M. (1998). Inferring pattern and process: maximum-likelihood implementation of a nonhomogeneous model of DNA sequence evolution for phylogenetic analysis. *Mol. Biol. Evol.* 15 871–879. 10.1093/oxfordjournals.molbev.a025991 9656487

[B42] Galtier N. Piganeau G. Mouchiroud D. Duret L. (2001). GC-content evolution in mammalian genomes: the biased gene conversion hypothesis. *Genetics* 159 907–911. 10.1038/35091126 11693127 PMC1461818

[B43] Gowri-Shankar V. Rattray M. (2007). A reversible jump method for Bayesian phylogenetic inference with a nonhomogeneous substitution model. *Mol. Biol. Evol.* 24 1286–1299. 10.1093/molbev/msm046 17347157

[B44] Haller B. C. Messer P. W. (2017). SLiM 2: flexible, interactive forward genetic simulations. *Mol. Biol. Evol.* 34 230–240. 10.1093/molbev/msw211 27702775

[B45] Hobolth A. Dutheil J. Y. Hawks J. Schierup M. H. Mailund T. (2011). Incomplete lineage sorting patterns among human, chimpanzee, and orangutan suggest recent orangutan speciation and widespread selection. *Genome Res.* 21 349–356. 10.1101/gr.114751.110 21270173 PMC3044849

[B46] International Human Genome Sequencing Consortium (2001). Initial sequencing and analysis of the human genome. *Nature* 412 860–921. 10.1038/35057062 11237011

[B47] Kent C. F. Minaei S. Harpur B. A. Zayed A. (2012). Recombination is associated with the evolution of genome structure and worker behavior in honey bees. *Proc. Natl. Acad. Sci. U.S.A.* 109 18012–18017. 10.1073/pnas.1208094109 23071321 PMC3497793

[B48] Kliman R. M. Hey J. (1994). The effects of mutation and natural selection on codon bias in the genes of *Drosophila*. *Genetics* 137 1049–1056. 7982559 10.1093/genetics/137.4.1049 PMC1206052

[B49] Kostka D. Hubisz M. J. Siepel A. Pollard K. S. (2012). The role of GC-biased gene conversion in shaping the fastest evolving regions of the human genome. *Mol. Biol. Evol.* 29 1047–1057. 10.1093/molbev/msr279 22075116 PMC3278478

[B50] Lachance J. Tishkoff S. A. (2014). Biased gene conversion skews allele frequencies in human populations, increasing the disease burden of recessive alleles. *Am. J. Hum. Genet.* 95 408–420. 10.1016/j.ajhg.2014.09.008 25279983 PMC4185123

[B51] Lartillot N. (2013). Interaction between selection and biased gene conversion in mammalian protein-coding sequence evolution revealed by a phylogenetic covariance analysis. *Mol. Biol. Evol.* 30 356–368. 10.1093/molbev/mss231 23024185

[B52] Lesecque Y. Glémin S. Lartillot N. Mouchiroud D. Duret L. (2014). The red queen model of recombination hotspots evolution in the light of archaic and modern human genomes. *PLoS Genet.* 10:e1004790 10.1371/journal.pgen.1004790 PMC4230742 25393762

[B53] Li C. Ortí G. (2007). Molecular phylogeny of Clupeiformes (Actinopterygii) inferred from nuclear and mitochondrial DNA sequences. *Mol. Phylogenet. Evol.* 44 386–398. 10.1016/j.ympev.2006.10.030 17161957

[B54] Liu L. Yu L. Pearl D. K. Edwards S. V. (2009). Estimating species phylogenies using coalescence times among sequences. *Syst. Biol.* 58 468–477. 10.1093/sysbio/syp031 20525601

[B55] Mallick S. Gnerre S. Muller P. Reich D. (2009). The difficulty of avoiding false positives in genome scans for natural selection. *Genome Res.* 19 922–933. 10.1101/gr.086512.108 19411606 PMC2675981

[B56] McCormack J. E. Faircloth B. C. Crawford N. G. Gowaty P. A. Brumfield R. T. Glenn T. C. (2012). Ultraconserved elements are novel phylogenomic markers that resolve placental mammal phylogeny when combined with species-tree analysis. *Genome Res.* 22 746–754. 10.1101/gr.125864.111 22207614 PMC3317156

[B57] Miller C. A. Hampton O. Coarfa C. Milosavljevic A. (2011). ReadDepth: a parallel R package for detecting copy number alterations from short sequencing reads. *PLoS ONE* 6:e16327 10.1371/journal.pone.0016327 PMC3031566 21305028

[B58] Montoya-Burgos J. I. Boursot P. Galtier N. (2003). Recombination explains isochores in mammalian genomes. *Trends Genet.* 19 128–130. 10.1016/S0168-9525(03)00021-0 12615004

[B59] Moriyama E. N. Powell J. R. (1997). Codon usage bias and tRNA abundance in *Drosophila*. *J. Mol. Evol.* 45 514–523. 10.1007/PL00006256 9342399

[B60] Mugal C. F. Weber C. C. Ellegren H. (2015). GC-biased gene conversion links the recombination landscape and demography to genomic base composition: GC-biased gene conversion drives genomic base composition across a wide range of species. *Bioessays* 37 1317–1326. 10.1002/bies.201500058 26445215

[B61] Nabholz B. Künstner A. Wang R. Jarvis E. D. Ellegren H. (2011). Dynamic evolution of base composition: causes and consequences in avian phylogenomics. *Mol. Biol. Evol.* 28 2197–2210. 10.1093/molbev/msr047 21393604 PMC3144382

[B62] Nagylaki T. (1983). Evolution of a finite population under gene conversion. *Proc. Natl. Acad. Sci. U.S.A.* 80 6278–6281. 10.1073/pnas.80.20.6278 6578508 PMC394279

[B63] Necsulea A. Popa A. Cooper D. N. Stenson P. D. Mouchiroud D. Gautier C. (2011). Meiotic recombination favors the spreading of deleterious mutations in human populations. *Hum. Mutat.* 32 198–206. 10.1002/humu.21407 21120948

[B64] Novembre J. A. (2002). Accounting for background nucleotide composition when measuring codon usage bias. *Mol. Biol. Evol.* 19 1390–1394. 10.1093/oxfordjournals.molbev.a004201 12140252

[B65] Pagani I. Liolios K. Jansson J. Chen I.-M. A. Smirnova T. Nosrat B. (2011). The Genomes OnLine Database (GOLD) v.4: status of genomic and metagenomic projects and their associated metadata. *Nucleic Acids Res.* 40 D571–D579. 10.1093/nar/gkr1100 22135293 PMC3245063

[B66] Pavlidis P. Jensen J. D. Stephan W. Stamatakis A. (2012). A critical assessment of storytelling: gene ontology categories and the importance of validating genomic scans. *Mol. Biol. Evol.* 29 3237–3248. 10.1093/molbev/mss136 22617950

[B67] Philippe H. Brinkmann H. Lavrov D. V. Littlewood D. T. J. Manuel M. Wörheide G. (2011). Resolving difficult phylogenetic questions: why more sequences are not enough. *PLoS Biol.* 9:e1000602 10.1371/journal.pbio.1000602 PMC3057953 21423652

[B68] Philippe H. Zhou Y. Brinkmann H. Rodrigue N. Delsuc F. (2005). Heterotachy and long-branch attraction in phylogenetics. *BMC Evol. Biol.* 5:50 10.1186/1471-2148-5-50 PMC1274308 16209710

[B69] Phillips M. J. Delsuc F. Penny D. (2004). Genome-scale phylogeny and the detection of systematic biases. *Mol. Biol. Evol.* 21 1455–1458. 10.1093/molbev/msh137 15084674

[B70] Plotkin J. B. Robins H. Levine A. J. (2004). Tissue-specific codon usage and the expression of human genes. *Proc. Natl. Acad. Sci. U.S.A.* 101 12588–12591. 10.1073/pnas.0404957101 15314228 PMC515101

[B71] Pollard K. S. Salama S. R. Lambert N. Lambot M.-A. Coppens S. Pedersen J. S. (2006). An RNA gene expressed during cortical development evolved rapidly in humans. *Nature* 443 167–172. 10.1038/nature05113 16915236

[B72] Pudlo P. Marin J.-M. Estoup A. Cornuet J.-M. Gautier M. Robert C. P. (2016). Reliable ABC model choice via random forests. *Bioinformatics* 32 859–866. 10.1093/bioinformatics/btv684 26589278

[B73] Ratnakumar A. Mousset S. Glémin S. Berglund J. Galtier N. Duret L. (2010). Detecting positive selection within genomes: the problem of biased gene conversion. *Philos. Trans. R. Soc. Lond. B Biol. Sci.* 365 2571–2580. 10.1098/rstb.2010.0007 20643747 PMC2935097

[B74] Risso D. Schwartz K. Sherlock G. Dudoit S. (2011). GC-content normalization for RNA-Seq data. *BMC Bioinformatics* 12:480 10.1186/1471-2105-12-480 PMC3315510 22177264

[B75] Rodríguez-Ezpeleta N. Brinkmann H. Roure B. Lartillot N. Lang B. F. Philippe H. (2007). Detecting and overcoming systematic errors in genome-scale phylogenies. *Syst. Biol.* 56 389–399. 10.1080/10635150701397643 17520503

[B76] Romiguier J. Cameron S. A. Woodard S. H. Fischman B. J. Keller L. Praz C. J. (2016). Phylogenomics controlling for base compositional bias reveals a single origin of eusociality in corbiculate bees. *Mol. Biol. Evol.* 33 670–678. 10.1093/molbev/msv258 26576851

[B77] Romiguier J. Ranwez V. Delsuc F. Galtier N. Douzery E. J. P. (2013a). Less is more in mammalian phylogenomics: AT-rich genes minimize tree conflicts and unravel the root of placental mammals. *Mol. Biol. Evol.* 30 2134–2144. 10.1093/molbev/mst116 23813978

[B78] Romiguier J. Ranwez V. Douzery E. J. P. Galtier N. (2010). Contrasting GC-content dynamics across 33 mammalian genomes: relationship with life-history traits and chromosome sizes. *Genome Res.* 20 1001–1009. 10.1101/gr.104372.109 20530252 PMC2909565

[B79] Romiguier J. Ranwez V. Douzery E. J. P. Galtier N. (2013b). Genomic evidence for large, long-lived ancestors to placental mammals. *Mol. Biol. Evol.* 30 5–13. 10.1093/molbev/mss211 22949523

[B80] Roux C. Fraïsse C. Romiguier J. Anciaux Y. Galtier N. Bierne N. (2016). Shedding light on the grey zone of speciation along a continuum of genomic divergence. *PLoS Biol.* 14:e2000234 10.1371/journal.pbio.2000234 PMC5189939 28027292

[B81] Roux C. Pauwels M. Ruggiero M.-V. Charlesworth D. Castric V. Vekemans X. (2012). Recent and ancient Signature of balancing selection around the S-Locus in *Arabidopsis halleri* and *A. lyrata*. *Mol. Biol. Evol.* 30 435–447. 10.1093/molbev/mss246 23104079 PMC3548311

[B82] Sheffield N. C. Song H. Cameron S. L. Whiting M. F. (2009). Nonstationary evolution and compositional heterogeneity in beetle mitochondrial phylogenomics. *Syst. Biol.* 58 381–394. 10.1093/sysbio/syp037 20525592

[B83] Smith J. M. Haigh J. (1974). The hitch-hiking effect of a favourable gene. *Genet. Res.* 23 23–35. 10.1017/S0016672300014634 4407212

[B84] Spencer C. C. A. (2006). Human polymorphism around recombination hotspots. *Biochem. Soc. Trans.* 34 535–536. 10.1042/BST0340535 16856853

[B85] Teeling E. C. Scally M. Kao D. J. Romagnoli M. L. Springer M. S. Stanhope M. J. (2000). Molecular evidence regarding the origin of echolocation and flight in bats. *Nature* 403 188–192. 10.1038/35003188 10646602

[B86] Urrutia A. O. (2003). The signature of selection mediated by expression on human genes. *Genome Res.* 13 2260–2264. 10.1101/gr.641103 12975314 PMC403694

[B87] Weber C. C. Boussau B. Romiguier J. Jarvis E. D. Ellegren H. (2014). Evidence for GC-biased gene conversion as a driver of between-lineage differences in avian base composition. *Genome Biol.* 15:549 10.1186/s13059-014-0549-1 PMC4290106 25496599

[B88] Williamson S. H. Hernandez R. Fledel-Alon A. Zhu L. Nielsen R. Bustamante C. D. (2005). Simultaneous inference of selection and population growth from patterns of variation in the human genome. *Proc. Natl. Acad. Sci. U.S.A.* 102 7882–7887. 10.1073/pnas.0502300102 15905331 PMC1142382

[B89] Yang Z. Nielsen R. (2002). Codon-substitution models for detecting molecular adaptation at individual sites along specific lineages. *Mol. Biol. Evol.* 19 908–917. 10.1093/oxfordjournals.molbev.a004148 12032247

